# A systematic review and meta-analysis of randomized controlled trials investigated the effects of melatonin supplementation on bone mineral density, quality of life, and sleep in menopausal women

**DOI:** 10.3389/fnut.2026.1687221

**Published:** 2026-01-29

**Authors:** Ji Du, Yong Tan

**Affiliations:** 1 Suzhou Hospital of Traditional Chinese Medicine, Suzhou, China; 2 Nanjing University of Chinese Medicine, Nanjing, China

**Keywords:** melatonin, menopause, bone mineral density, sleep quality, menopausal symptoms, meta-analysis

## Abstract

**Objective:**

This study evaluated the impact of melatonin supplementation on bone mineral density (BMD), sleep quality, menopausal symptoms, mood, sexual function, serum insulin levels, and body mass index in menopausal women.

**Evidence review:**

A systematic literature review and meta-analysis were conducted using PubMed/MEDLINE, Embase, and Web of Science (2015–2024), following PRISMA guidelines. The risk of bias was assessed with the Cochrane tool.

**Findings:**

Analysis of 7 groups (497 participants) indicated that melatonin may increase bone mineral density (BMD), particularly in the femoral neck, based on two randomized controlled trials (RCTs). However, high heterogeneity prevented pooled statistical analysis. No significant improvements were observed in sleep quality, menopausal symptoms, anxiety, depression, sexual function, BMI, or insulin levels. Side effects were similar across groups.

**Conclusion:**

Available evidence suggests that melatonin-containing supplements may be associated with improved BMD in menopausal women, but the independent effect of melatonin and an optimal dose remain unclear due to heterogeneity in interventions and the prevalent use of combination therapies. For other outcomes (sleep, menopausal symptoms, mood, sexual function, BMI, and insulin), the evidence is currently inconclusive. Further large-scale RCTs are needed to confirm these findings.

## Introduction

Menopause generally occurs between ages 45 and 56. During menopause, vasomotor symptoms like hot flashes and night sweats affect 50 to 75% of women, while over half experience urogenital symptoms, also known as menopausal urogenital syndrome (GSM) (1). All women will experience menopause. Alterations in reproductive hormones lead to symptoms that define this transitional phase. There are three distinct phases in the menopausal transition: perimenopause, menopause, and post-menopause. Symptoms related to each phase can negatively influence women’s mental and physical performance in daily life (2).

Hormone therapy effectively alleviates menopausal symptoms like VMS but may adversely affect the cardiovascular system, including causing thromboembolic events (3, 4). Additionally, it is not suitable for certain groups, such as individuals who have survived breast cancer and those with a familial history of the condition (5). Therefore, there is a pressing demand for a non-hormonal replacement therapy that is both effective and well-tolerated.

An indoleamine hormone called melatonin, or N-acetyl-5-methoxytryptamine, is synthesized by the pineal gland. Melatonin is present in various tissues, including the female reproductive system, where it plays a key role in mediating the suprachiasmatic nucleus (SCN) to regulate the circadian rhythm of peripheral organs (6). Recent studies have highlighted its diverse functions, particularly as a strong antioxidant, an immune-active component, and a regulator of mitochondria (7).

Melatonin potentially influences menopausal physiology through multiple mechanisms. Regarding sleep, melatonin may contribute to the regulation of sleep patterns during menopause by modulating sleep architecture (8). Concerning bone metabolism, melatonin is posited to facilitate the differentiation of bone marrow mesenchymal stem cells (BMSCs) into osteoblasts, elevate serum levels of alkaline phosphatase (ALP) and osteocalcin, and enhance the mRNA expression of osteogenic-related genes such as Runx2 and Sp7, thereby ameliorating bone metabolism (9). Furthermore, melatonin may indirectly influence the physiological status of menopausal women by modulating the immune system and oxidative stress. This includes regulating immune cell numbers and cytokine expression, decreasing the secretion of inflammatory factors, enhancing immune function, and mitigating the detrimental effects of inflammatory damage on the body (10).

The advantages of melatonin for women experiencing menopause have been recognized (11). Few trials and two meta-analyses have evaluated how effective melatonin is for peri-menopausal and post-menopausal women (12, 13). The effectiveness of melatonin in managing menopausal and postmenopausal women is determined through this systematic review and meta-analysis of controlled trials.

### *A priori* hypotheses

Prior to data extraction and analysis, we formulated the following directional hypotheses based on preclinical evidence and prior clinical observations: (1) Melatonin supplementation would improve bone mineral density (BMD) in menopausal women; (2) Melatonin would improve sleep quality and reduce the severity of menopausal symptoms; (3) Melatonin would have a beneficial or neutral effect on mood (anxiety and depression), sexual function, body mass index (BMI), and serum insulin levels.

## Methods

### Search criteria

Following PRISMA guidelines (14), we conducted a comprehensive review of literature using PubMed/MEDLINE, Embase, and Web of Science from 2015 to October 2024. This is due to a significant rise in published data on melatonin in menopausal women since 2015. Relevant keywords for this study include “menopause,” “post-menopause,” “peri-menopause,” “climacteric,” “melatonin” and “N-acetyl serotonin.” A manual search of reference lists was also conducted to identify pertinent literature.

### Criteria for inclusion and exclusion

For this systematic review and meta-analysis, two reviewers working in pairs independently selected studies according to predefined inclusion criteria. The eligibility criteria were established in accordance with the PICOS framework-encompassing Population, Intervention, Comparison, Outcomes, and Study Design-as outlined in Table 1. Inclusion criteria for the studies were: (I) publication date between 2015 and 2024, and (II) randomized controlled trials that evaluated melatonin supplementation’s efficacy in perimenopausal or postmenopausal women, and (III) studies published in English.

**Table 1:** Study inclusion criteria according to PICOS framework.

Element	Inclusion criteria
*P* (Population)	Perimenopausal or postmenopausal women.
*I* (Intervention)	Oral melatonin supplementation, regardless of dosage, formulation, or treatment duration (as either a monotherapy or a component of a combination supplement).
*C* (Comparator)	Placebo.
*O* (Outcomes)	*Primary outcomes*: Bone mineral density (BMD), measured by dual-energy X-ray absorptiometry (DXA) or quantitative computed tomography (QCT). *Secondary outcomes*: Sleep quality (e.g., Pittsburgh Sleep Quality Index [PSQI], Insomnia Severity Index [ISI]). Menopausal symptoms (e.g., Menopause-Specific Quality of Life [MENQOL], Kupperman Index [KI]). Mood status: anxiety (e.g., Hamilton Anxiety Rating Scale [HARS]) and depression (e.g., Beck Depression Inventory [BDI]). Sexual function (Female Sexual Function Index [FSFI]). Body mass index (BMI). Serum insulin levels. Safety and adverse events.
*S* (Study design)	Randomized Controlled Trials (RCTs).

Studies were excluded if they were non-randomized controlled trials, not published in English, or lacked accessible full text. Initially, titles and abstracts were screened by researchers to filter out studies that did not adhere to the inclusion criteria. Subsequently, the remaining studies underwent a detailed independent review, and the worklists from two authors were compared. When disagreements arose, all researchers participated in discussions to reach a consensus.

**Figure 1:**
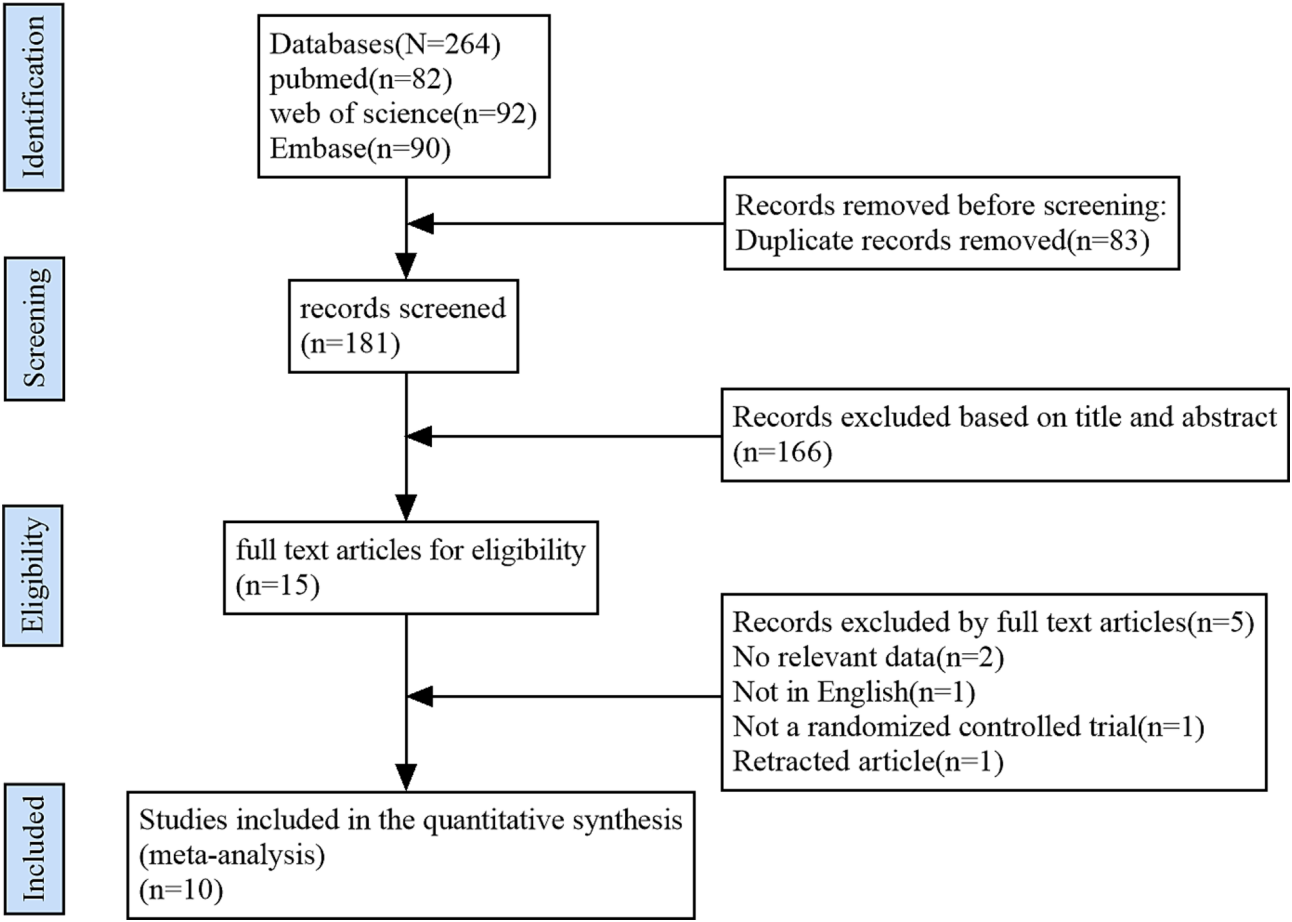
Flowchart showing the process of selecting studies.

The study selection process is depicted in Figure 1 using the PRISMA flow diagram.

### Study selection procedure

Following the elimination of duplicates, two reviewers assessed the titles and abstracts. Studies not meeting eligibility criteria were systematically excluded, and the first author organized the downloaded full texts by publication year. The two authors independently assessed the articles and selected those that satisfied the inclusion criteria.

### Data extraction

We extracted data using specific forms created for this review (see Table 2). The forms included: (1) details about the article (author, year of publication, country); (2) specifics of the study (location, design, number of participants, participant information); (3) medication details (dose, frequency, duration); and (4) outcome data (measurement tools). For continuous outcomes, we obtained the mean, standard deviation (SD), and sample size for both baseline and post-intervention evaluations. In the case of dichotomous outcomes, we collected data on the number of events and the total sample size within each group. Additionally, we recorded the specific time points at which outcomes were measured, such as the conclusion of treatment or particular follow-up visits. Furthermore, the specific scales or instruments employed to assess each outcome were meticulously documented. To reduce potential bias, two researchers independently conducted the literature review, selected articles, extracted data, and assessed the risk of bias. Whenever conflicts arose, we consulted other researchers and settled our disagreements through discussion. To optimize melatonin’s impact, studies with multiple outcome measurements included only the final time point results. The included RCTs were assessed using the Cochrane risk of bias tool (version 2.0, RoB 2). We conducted the RoB assessment for the primary outcome of interest at the final follow-up time point for each included study. Through discussions and a review process, all disputes were resolved.

**Table 2:** Features of the 10 articles included (7 cohorts).

Author	Publication year	Country	Study design	Sample size	Study arms	Experimental group	Control group	Dosage form	Ingredient type	Durition	Melatonin dose/d	Age (mean±SD)	Menopause status	Co-existing disease	Follow-up periods (since treatment onset)	Outcomes	Measurements
Amstrup et al (16)	2015	Denmark	RCT	81	Melatonin (*n* = 40) Placebo (*n* = 41)	1 or 3 mg p.o. at bedtime +800 mg calcium/d + 20 ug vitamin D3/d	Placebo+800 mg calcium/d + 20 ug vitamin D3/d	tablet	multiple	12 mon	1 or 3 mg	EG:62.4 ± 3.5 CG:62.9 ± 4.7	Postmenopausal	Osteoporosis	3,6,9, and 12 mon	BMD	DXA, HR-pQCT
Amstrup et al (17)	2015	Denmark	RCT	81	Melatonin (*n* = 40) Placebo (*n* = 41)	1 or 3 mg melatonin nightly+800 mg calcium/d + 20 ug vitamin D3/d	Placebo+800 mg calcium/d +20 ug vitamin D3/d	tablet	multiple	12 mon	1 or 3 mg	EG:62.4 ± 3.5 CG:62.9 ± 4.7	Postmenopausal	Osteopenia	12 mon	Quality of life or sleep, muscle function	PSQI, PAS, SF-36, WHO-5
Chojnacki et al (18)	2015	Poland	RCT	64	Fluoxtine+Melatonin (*n* = 34) Fluoxtine (*n* = 30)	20 mg fluoxetine in the morning+5 mg melatonin in the evening	20 mg fluoxetine in the morning +placebo (in the evening)	Not mentioned	multiple	6 mon	5 mg	EG:57.9 ± 5.50 CG:56.10 ± 5.8	Postmenopausal	Overweight with increased appetite	week 2, 4, 8, 12, 16, 20 and 24	Anxiety, depression, sleep quality	BMI, HARS, BDI, ISI
Amstrup et al (19)	2016	Denmark	RCT	81	Melatonin (*n* = 40) Placebo (*n* = 41)	1 or 3 mg melatonin nightly +800 mg calcium/d + 20 ug vitamin D3/d	Placebo+800 mg calcium/d + 20 ug vitamin D3/d	Not mentioned	multiple	12 mon	1 or 3 mg	EG:62.4 ± 3.5 CG:62.9 ± 4.7	Postmenopausal	Osteopenia	12 mon	Body composition, biochemistry	DXA, plasma insulin
D’Anna et al (20)	2017	Italy	RCT	32	Melatonin (*n* = 16) Placebo (*n* = 16)	2 g/d myo-Ins + 3 g/d melatonin before sleeping	myo-Ins 2 g twice a day	Not mentioned	multiple	6 mon	3 g	EG:49.1 ± 1.7 CG:48.7 ± 1.5	Perimenopausal	Healthy	6 mon	Serum insulin and serum thyroid profile	BMI, serum insulin
Maria et al (21)	2017	USA	RCT	22	MSDK (*n* = 11) Placebo (*n* = 11)	2 MSDK capsules (5 mg melatonin), at bedtime	Placebo (contained plant fiber)	capsule	multiple	12 mon	5 mg	EG:57 ± 1.41 CG:60 ± 1.73	Postmenopausal	Osteopenia	12 mon	BMD, mood and sleep quality	DXA, MENQOL; STAI; PSS; CES-D
Parandavar et al (22)	2017	Iran	RCT	199	Melatonin (*n* = 98) Placebo (*n* = 101)	3 mg melatonin per night at 6–9 p.m.	Placebo (lactose, avicel, and magnesium stearate)	tablet	single	3 mon	3 mg	EG:52.85 ± 4.26 CG:53.39 ± 4.22	Postmenopausal	Sexual dysfunction	1,2, and 3 mon	sexual function	FSFI
Chojnacki et al (23)	2018	Poland	RCT	60	Melatonin (*n* = 30) Placebo (*n* = 30)	3 mg melatonin at the morning and of 5 mg melatonin at the bedtime	Placebo	Not mentioned	single	12 mon	8 mg	EG:57.3 ± 6.4 CG:56.2 ± 4.1	Postmenopausal	Healthy	2, 4, 6, 8, 10 and 12 mon	Menopausal symptoms	BMI, KI
Parandavar et al (24)	2018	Iran	RCT	199	Melatonin (*n* = 98) Placebo (*n* = 101)	3 mg melatonin every evening 6–9 p.m.	Placebo	tablet	single	3 mon	3 mg	EG:52.8 ± 29.4 CG:53.63 ± 4.1	Menopausal	Healthy	3 mon	Lipid profile, complications	Side effects
Amstrup et al (25)	2024	Denmark	RCT	39	Melatonin (*n* = 21) Placebo (*n* = 20)	10 mg melatonin per day	Placebo	Not mentioned	single	3 mon	10 mg	EG:63 CG:64	Postmenopausal	Healthy	3 week and 3 mon	Sleep quality	PSQI

EG, Experimental Group; CG, Control Group; MSDK, Contained in two capsules, the MSDK formulation comprised 5 mg of melatonin, 450 mg of strontium (citrate), 2000 IU of vitamin D3, and 60 μg of vitamin K2; BMD, bone mineral density; DXA, dual X-ray absorptiometry; HR-pQCT, high-resolution peripheral QCT; PSQI, Pittsburgh Sleep Quality Index; PAS, Physical Activity Scale; SF-36, Short Form questionnaire 36; WHO-5, WHO-Five Well Being Index; BMI, body mass index; HARS, Hamilton anxiety rating scale; BDI, Beck depression scale; ISI, insomnia severity index; MENQOL, Menopause specific Quality Of Life; STAI, State and Trait Anxiety Inventory; PSS, Perceived Stress Scale; CES-D, Center for Epidemiologic Studies Depression; FSFI, Sexual Function Index; KI, Kupperman Index.

### Analysis of statistics

PRISMA guidelines were followed in conducting the systematic review. For the limited number of studies that reported outcomes as medians and interquartile ranges instead of means and standard deviations, we estimated the means and standard deviations using the method described by Wan et al. (15). For studies lacking standard deviations (SDs) for continuous outcomes, we endeavored to contact the corresponding authors to acquire the original data. In terms of outcome data selection, when change-from-baseline data accompanied by a measure of variance were not explicitly reported, we utilized the final (post-intervention) values for the meta-analysis, given the availability of both pre- and post-intervention data. We recorded the scores of change from baseline to outcomes to clearly demonstrate the improvement associated with melatonin.

The standardized mean difference (SMD) was computed using Cohen’s analysis for studies with different scales or self-reported outcomes to standardize the results. Calculate the weighted mean difference (WMD) for body mass index (BMI). We employed a random-effects model for meta-analyses to account for anticipated clinical and methodological heterogeneity among the included studies, as it provides a more conservative and generalizable estimate of the mean effect. Heterogeneity across studies was assessed using the I^2^ statistic, which quantifies the percentage of total variation across studies that is due to heterogeneity rather than chance. An I^2^ value greater than 50% was considered to indicate substantial heterogeneity. Substantial heterogeneity is indicated by an I^2^ value exceeding 50%, with significance determined by a *p*-value under 0.1. The statistical analyses were performed with Review Manager version 5.3.0, provided by the Cochrane Collaboration, UK.

## Results

### Study description

In our preliminary search, we identified 264 publications. Once duplicates were removed, we examined the titles and abstracts of 181 articles and excluded 166 based on our criteria. After downloading the complete texts of the last 15 selected studies and meticulously reviewing the information, we ultimately incorporated 10 articles, representing 7 randomized controlled trials (RCTs), into this meta-analysis (16–25). The rationale for the exclusion of the final 10 articles is illustrated in Figure 1.

We compiled the fundamental characteristics of the seven RCTs incorporated in this analysis, which collectively involved 497 participants. The studies, published from 2015 to 2024, had sample sizes ranging from 22 participants (21) to 240 participants (22, 24). Participant ages averaged between 48.7 and 62.9 years. Five studies specifically targeted postmenopausal women (20), while one included perimenopausal women and another focused on menopausal women (24). However, some studies lacked clear definitions and criteria regarding menopause. The participant pool was not exclusively composed of healthy women; women with osteopenia (16, 17, 19, 21), overweight (18), and sexual dysfunction (22) also participated in three of the RCTs (Table 1).

The analyzed randomized controlled trials (RCTs) (refer to Table 1) involved administering melatonin with variations in duration (3 to 12 months), dosage (1 mg to 3 g), and timing (6 p.m. to bedtime). Melatonin was combined with other pharmaceutical agents in three research studies: in one case, 20 mg of fluoxetine was administered (18), another included 2 g of myo-inositol (20), and the third included 450 mg of strontium citrate, 2,000 IU of vitamin D3, and 60 mg of vitamin K_2_ (MSDK) in their regimen (21) (refer to Table 1).

Table 1 presents the results from the RCTs analyzed in this meta-analysis. Measurements are also provided. Three randomized controlled trials evaluated sleep quality: the Pittsburgh Sleep Quality Index was employed in one study (25), whereas the Insomnia Severity Index was used by Chojnacki et al. (18). Three randomized controlled trials used the WHO-Five Well-Being Index, Kupperman Index, and Menopause-Specific Quality of Life to assess menopause symptoms.

The Beck Depression Scale (18) and the Center for Epidemiologic Studies (21) were used to assess mood status for depression, while anxiety was measured using the Hamilton Anxiety Rating Scale (18) and the State–Trait Anxiety Inventory (21). Additionally, data on altered serum insulin levels (19, 20) and BMI (18, 19, 21) were gathered from four RCTs (refer to Table 1).

### Evaluation of the risk of bias in the studies included

**Figure 2:**
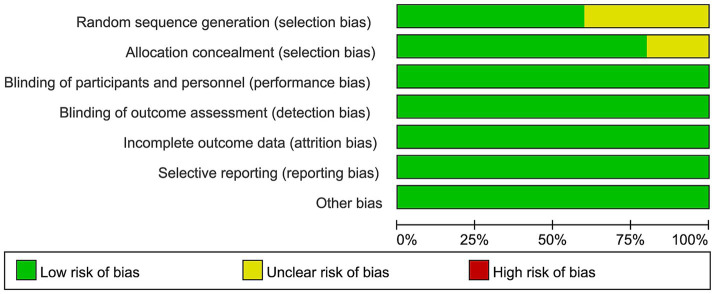
The graph depicting bias risk.

Regarding bias risk in the studies included, four of the ten RCTs showed uncertain risks related to random sequence generation because the randomization methods were not clearly described. The other six studies were judged to carry a low risk of bias. Two of the ten RCTs were noted for unclear risks in allocation concealment stemming from insufficient descriptions of the allocation concealment processes. All ten RCTs showed a low risk of bias regarding participant and personnel blinding, outcome assessment blinding, completeness of outcome data, selective reporting, and other possible bias sources (Figure 2).

### Results of meta-analysis

#### Primary outcomes

##### Bone mineral density

To measure BMD, Amstrup et al. (16) applied dual-energy X-ray absorptiometry and quantitative computed tomography. Similarly, Maria et al. (21) measured bone mineral density using DXA. Maria’s findings were derived from a comparison between combination therapy, which included a compound formulation containing melatonin, and a placebo. Due to heterogeneity in both intervention and methodology between these two studies, the BMD data cannot be merged, leading to the discontinuation of the statistical analysis. Amstrup et al. (16) reported an increase in femoral neck aBMD (DXA) in both the 1 mg (*p* = 0.55) and 3 mg (*p* < 0.01) melatonin groups, alongside a rise in lumbar spine vBMD (QCT) in the 3 mg group (*p* = 0.04). Significantly, the rise in aBMD of the femoral neck showed a dose-dependent correlation (*p* < 0.05). The study by Maria et al. (21) revealed a statistically significant difference between the MSDK and placebo groups in the left femoral neck (*p* < 0.001) and lumbar spine (L1-L4) (*p* = 0.021). Both studies reported increased bone density values in the melatonin groups compared to controls, with statistically significant improvements specifically at the femoral neck in the 3 mg group in the Amstrup et al. trial (*p* < 0.01) and in the MSDK combination therapy group in the Maria et al. trial (*p* < 0.001).

#### Secondary outcomes

**Figure 3:**
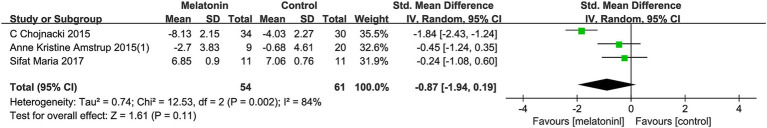
Displays forest plots illustrating the differences in sleep quality between melatonin and control groups; SD stands for standard deviation, IV for inverse variance, CI for confidence interval, and df for degrees of freedom.

**Figure 4:**
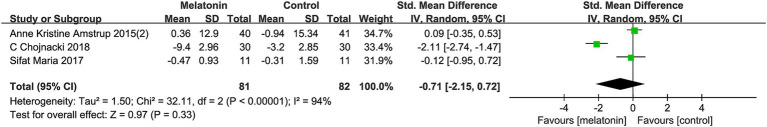
Displays forest plots illustrating the differences in menopausal symptoms between melatonin and control groups; SD stands for standard deviation, IV for inverse variance, CI for confidence interval, and df for degrees of freedom.

**Figure 5:**
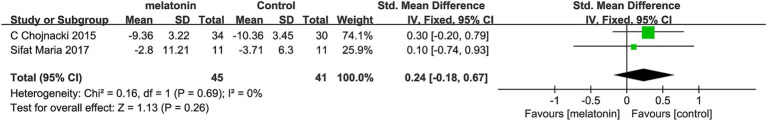
Displays forest plots illustrating the differences in anxiety between melatonin and control groups; SD stands for standard deviation, IV for inverse variance, CI for confidence interval, and df for degrees of freedom.

**Figure 6:**
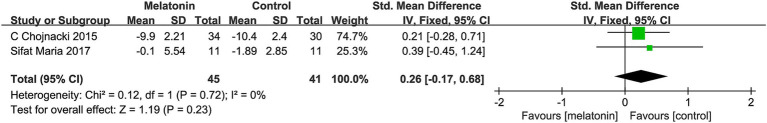
Displays forest plots illustrating the differences between melatonin and control groups in depression; SD stands for standard deviation, IV for inverse variance, CI for confidence interval, and df for degrees of freedom.

**Figure 7:**
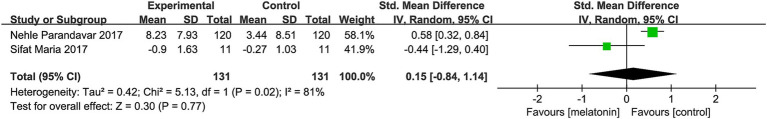
Displays forest plots illustrating the differences in sexual function between melatonin and control groups; SD stands for standard deviation, IV for inverse variance, CI for confidence interval, and df for degrees of freedom.

**Figure 8:**
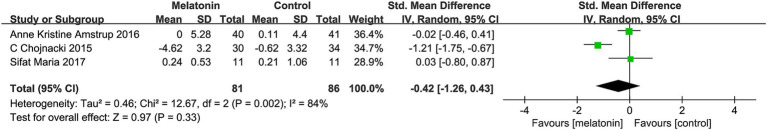
Displays forest plots illustrating the differences in BMI between melatonin and control groups; SD stands for standard deviation, IV for inverse variance, CI for confidence interval, and df for degrees of freedom.

**Figure 9:**
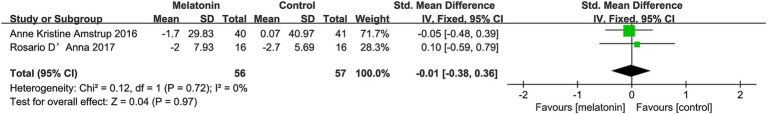
Displays forest plots illustrating the differences between melatonin and control in INS; SD stands for standard deviation, IV for inverse variance, CI for confidence interval, and df for degrees of freedom.

**Figure 10:**
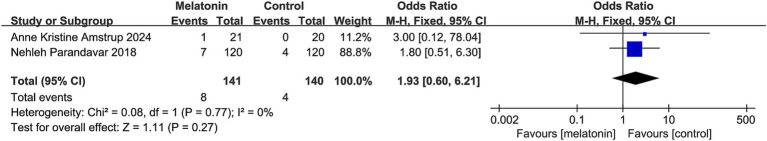
Forest plot for the comparison of adverse events (melatonin vs. control). Results are presented as odds ratios (ORs) with 95% confidence intervals (CIs), pooled using a random-effects model.

In three randomized controlled trials, melatonin treatment showed no improvement in sleep quality (SMD −0.87; 95% CI, −1.94 to 0.19, *p* = 0.11, I^2^ = 84%) (Figure 3) or menopause symptoms (SMD −0.71; 95% CI, −2.15 to 0.72, *p* = 0.33, I^2^ = 94%) (Figure 4). The analysis showed no significant differences in anxiety (SMD 0.24; 95% CI, −0.18 to 0.67, *p* = 0.26, I^2^ = 0%) (Figure 5), depression (SMD 0.26; 95% CI, −0.17 to 0.68, *p* = 0.236, I^2^ = 0%) (Figure 6), or sexual function (SMD 0.15; 95% CI, 0.84 to 1.14, *p* = 0.77, I^2^ = 81%) (Figure 7) between women who received melatonin treatment and those who were administered a placebo. According to the pooled analysis, there were no significant changes in BMI (SMD −0.42; 95% CI, −1.26 to 0.43, *p* = 0.33, I^2^ = 84%) (Figure 8) or insulin levels (SMD −0.01; 95% CI, −0.38 to 0.36, *p* = 0.97, I^2^ = 0.0%) (Figure 9) between the melatonin and placebo groups following the intervention. Concerning side effects, just two studies (24, 25) supplied relevant data. There was no significant variation in side effect rates between those taking melatonin and those on a placebo (OR 1.93; 95% CI, 0.60–6.21; *p* = 0.27; I^2^ = 0%) (Figure 10).

## Discussion

Ten randomized controlled trials (RCTs) were examined to understand how supplemental melatonin affects bone mineral density (BMD), quality of life, and sleep in menopausal women. The available findings from two RCTs suggest that melatonin-containing supplements may be associated with enhanced BMD in menopausal women, although the evidence remains limited and derived from combination therapies. The study found no significant differences in sleep quality, menopausal symptoms, mood status, sexual function, BMI, or insulin levels. This meta-analysis demonstrates varying levels of heterogeneity, from low to high.

Currently, there are no definitive guidelines regarding the optimal dosage and frequency for using melatonin. In this meta-analysis, the RCTs mainly involved oral supplements taken at before sleep, with one study also including morning tablets (23). The dosages ranged from 1 mg to 3 g, with 3 mg being the most frequently used.

Amstrup et al. (16, 17) justified their selection of 1 mg and 3 mg doses to minimize the risk of discomfort among participants. In various European nations, melatonin is available only by prescription, with a suggested nightly dosage of 2 mg as a sleep aid. The duration of interventions in the studies analyzed varied from 3 to 12 months. According to Amstrup et al. (16), taking 3 mg of melatonin daily can improve bone density in the femoral neck, a common fracture site (26), indicating that melatonin might be useful in preventing fractures. Nonetheless, the precise mechanism by which melatonin mitigates fracture risk remains elusive. Amstrup et al. (16) propose that the observed increase in mineralization within the melatonin-treated group may be partially attributed to reduced urinary calcium excretion. Research indicates that melatonin helps protect against osteoporosis by: (1) stimulating osteoblast activity and bone formation (27), (2) inhibiting osteoclast differentiation and activity, thus reducing bone resorption via the RANKL pathway (28), and (3) eliminating free radicals, reducing oxidative damage, and suppressing pro-inflammatory cytokines in bone tissue (29).

### Reconciliation of mechanistic interpretations and limitations of combination therapy

The observed improvements in bone mineral density (BMD) are consistent with preclinical mechanistic evidence indicating that melatonin promotes osteoblast differentiation, inhibits osteoclast activity, and reduces oxidative stress in bone tissue. Nevertheless, all randomized controlled trials (RCTs) focusing on BMD have employed combination therapies (melatonin in conjunction with calcium, vitamin D, or strontium), thereby raising questions regarding the independent effect of melatonin. This apparent contradiction can be resolved by acknowledging the synergistic nature of these interventions: micronutrients such as calcium and vitamin D provide essential substrates for bone formation, while melatonin modulates bone cell activity, resulting in a complementary effect that enhances BMD improvements. Preclinical studies corroborate that melatonin’s osteogenic effects are augmented in the presence of sufficient calcium and vitamin D, thereby underscoring the clinical significance of combination regimens. Importantly, the absence of data on melatonin monotherapy does not negate its mechanistic role but rather highlights a gap in the current body of evidence. Future three-arm randomized controlled trials (RCTs) comparing melatonin alone, combination therapy, and placebo are necessary to elucidate the independent effect of melatonin on bone mineral density (BMD) enhancement. In the absence of such data, the clinical benefits observed from melatonin-containing combination supplements are both biologically plausible and clinically significant. These findings are consistent with preclinical mechanisms and the real-world practice of co-prescribing nutrients that support bone health.

This meta-analysis encompassed perimenopausal and postmenopausal women, who experience fluctuations and alterations in sex hormone levels during these stages. The connection between melatonin and sex hormones is not yet well-defined in current research. It’s unclear if the alleviation of menopausal symptoms due to melatonin is linked to alterations in sex hormone levels. Further investigation is required due to this uncertainty. While our meta-analysis found no significant overall effect of melatonin on sleep quality, it is noteworthy that some individual studies included women with diagnosed sleep disorders and reported positive outcomes. This indicates that the initial sleep status may influence the effectiveness of melatonin, a hypothesis that could not be examined through subgroup analysis due to the limited sample size. This suggests that future research should directly investigate whether baseline sleep status moderates melatonin’s efficacy.

Additionally, the study reveals that exogenous melatonin intake does not significantly alleviate anxiety and depression in postmenopausal women. Prior studies have linked melatonin secretion and its circadian rhythm to mood disorders, suggesting that reduced melatonin levels at bedtime may hinder sleep onset (30).

Furthermore, the null effect on sexual function observed in our meta-analysis suggests that, even if preclinical data suggest melatonin may modulate sexual arousal thresholds, this does not translate to a measurable improvement in self-reported sexual function scores in postmenopausal women within the studied contexts. This limitation may arise from both the inherent properties of melatonin and the complexities involved in scoring sexual function scales. Future research should explore the potential of melatonin as a health supplement for addressing sexual dysfunction in postmenopausal women.

Amstrup et al. (19) previously found that melatonin can reduce fat mass and potentially increase lean mass in women who are middle-aged. Our meta-analysis found no significant effect of melatonin on BMI or insulin levels. It is plausible that the considerable heterogeneity in melatonin dosages (1 mg to 3 g) and the inclusion of combination supplements obscured any potential dose-dependent or formulation-specific metabolic effects. Future research should explore the use of melatonin in treating metabolic disorders like polycystic ovary syndrome. Nonetheless, extensive clinical randomized trials are required to confirm these results.

The interpretation of the non-significant findings related to the secondary outcomes-namely sleep, menopausal symptoms, mood, sexual function, BMI, and insulin-necessitates caution. The broad confidence intervals observed in these analyses suggest imprecision, likely attributable to limited sample sizes and substantial heterogeneity. Consequently, these findings should not be construed as definitive evidence of no effect (proof of ineffectiveness); rather, they should be viewed as inconclusive or indicative of insufficient evidence to detect a meaningful difference, should one exist. To ascertain whether melatonin exerts a true null effect or a small but clinically significant effect on these outcomes, larger and more homogeneous trials with enhanced statistical power are required.

When interpreting the non-significant results for secondary outcomes such as sleep quality, menopausal symptoms, mood, sexual function, BMI, and insulin levels, it is essential to differentiate between the absence of evidence for an effect and evidence indicating a lack of effect. The broad confidence intervals and considerable heterogeneity (I^2^ > 50%) observed in these meta-analyses suggest imprecision and inconsistency within the current body of evidence. Therefore, these findings should be more accurately characterized as inconclusive or indicative of insufficient data, rather than as definitive evidence that melatonin exerts no effect on these outcomes. To ascertain the existence of any true effect, larger, more homogeneous, and adequately powered randomized controlled trials are necessary.

### Strengths and limitations

This extensive review and meta-analysis have multiple strengths. The findings’ accuracy was improved by having two researchers independently select the articles. The literature from post-2015 was comprehensively reviewed to assess how melatonin consumption affects bone mineral density, quality of life, and sleep in menopausal women. Thirdly, the ten randomized controlled trials (RCTs) included in the study were evaluated as being of good quality, with none deemed high-risk.

Our meta-analysis is subject to several limitations. Firstly, there was considerable heterogeneity in the sample sizes of the included studies, which ranged from small-scale trials (*n* < 50) to larger randomized controlled trials (RCTs). Although this heterogeneity could theoretically impact the precision of estimates from individual studies, we implemented several methodological safeguards to mitigate this issue: (1) a random-effects model was utilized to account for between-study variance; and (2) the methodological quality of all included studies was rigorously evaluated using the Cochrane Risk of Bias tool. Despite these measures, the results should be interpreted with caution. Future research should include large-scale, well-designed RCTs to confirm these findings. Furthermore, the implementation of standardized methodologies, such as consistent dosing and uniform measurement tools, alongside larger homogeneous sample sizes, will facilitate subgroup analyses based on key moderators, including baseline sleep status, menopausal phase, and dosage. This approach aims to elucidate the sources of heterogeneity. Secondly, we did not impose a minimum treatment duration or dose threshold as inclusion criteria. This decision aimed to encompass the full spectrum of clinical practice and prevent the exclusion of potentially informative studies, particularly given the limited number of available randomized controlled trials (RCTs). Although this inclusive approach broadened the scope of our evidence synthesis, it inherently restricted our capacity to draw definitive conclusions regarding the efficacy of specific treatment durations or to determine a minimum effective dose. As a result, our findings reflect an average effect across a diverse array of intervention protocols. Future research should explore the use of melatonin in treating metabolic disorders like polycystic ovary syndrome. Nonetheless, extensive clinical randomized trials are required to confirm these results. Thirdly, despite the high quality of RCTs in BMD research, the quantity and sample size of these trials remain inadequate. All clinical trials on BMD used combined interventions (e.g., melatonin with calcium, vitamin D, or strontium), preventing the assessment of melatonin’s independent effect. Future studies should focus on isolating melatonin’s specific contribution. Fourthly, the utilization of diverse measurement tools and standards across studies introduces inconsistencies that impede the integration and comparison of findings, thereby complicating the comprehensive assessment of melatonin’s efficacy. Future research should endeavor to standardize methodologies, ensure larger and more homogeneous sample sizes, and incorporate long-term follow-up studies to address these limitations and enhance the understanding of melatonin’s role in clinical practice. Fifthly, we did not conduct assessments for publication bias, such as funnel plots or Egger’s test, due to the limited number of studies available for each outcome (most comprising five or fewer randomized controlled trials), which is inadequate for the reliable detection of asymmetry. Although all included randomized controlled trials were registered and peer-reviewed, the possibility of underreporting negative findings cannot be entirely ruled out. Future meta-analyses with a larger number of included studies should incorporate these assessments. Furthermore, as detailed in the ‘Heterogeneity of Melatonin Preparations’ section, the significant variations in melatonin dosage, formulation, and the common use of combination therapies across the included studies limit our ability to draw definitive conclusions this regarding an optimal dosage or the independent efficacy of melatonin monotherapy.

### Heterogeneity of melatonin preparations

The included randomized controlled trials (RCTs) demonstrated significant clinical heterogeneity in the formulation of melatonin supplements. As outlined in Table 1, the interventions varied in dosage form, melatonin strength (ranging from 1 mg to 3 g), and compositional type. A substantial number of trials administered melatonin as part of a combination therapy, most notably with calcium and vitamin D3 for bone health (16, 17, 19, 21), or with other active compounds such as fluoxetine (18) and myo-inositol (20). For the secondary outcomes of this meta-analysis—including sleep quality, menopausal symptoms, mood, sexual function, and metabolic parameters—the influence of these adjunct ingredients is likely minimal. Micronutrients such as calcium and vitamin D are not known to exert direct, significant effects on the neurophysiological pathways governing these specific outcomes. Therefore, the overall null findings observed for these endpoints are unlikely to be substantially affected by the heterogeneity in composition. In contrast, the interpretation of the primary outcome, bone mineral density (BMD), is inherently confounded by the prevalent use of combination regimens in the relevant trials.

These methodological considerations have a direct impact on clinical practice. The null findings for most outcomes suggest that melatonin, as it is typically supplemented-often in combination with other agents-cannot be recommended as a reliable monotherapy for alleviating common menopausal symptoms such as poor sleep, vasomotor symptoms, or low mood. Although there is potential benefit for bone health, this is currently indistinguishable from the effects of co-administered nutrients, such as calcium and vitamin D. Therefore, definitive conclusions regarding the independent therapeutic value of melatonin, and its evidence-based clinical application, must await future trials specifically designed to isolate its effects. Ultimately, the clinical utility of melatonin depends on patient-specific factors and the context of the intervention.

The safety of melatonin. Our meta-analysis indicated that melatonin supplementation is well-tolerated in menopausal women for short-term use (≤12 months), exhibiting an adverse event profile similar to that of a placebo. Nevertheless, this conclusion necessitates cautious interpretation in light of recent evidence concerning the long-term safety of melatonin use. A large-scale observational study, presented at the 2025 American Heart Association (AHA) Scientific Sessions by Li and Van Horn (31), has raised substantial concerns about the cardiovascular safety associated with long-term prescription melatonin use (defined as use for 12 months or more). The study identified a significant association between long-term melatonin use and an increased risk of incident heart failure (hazard ratio [HR] 1.89), heart failure hospitalization (HR 3.44), and all-cause mortality (HR 2.09) among patients with chronic insomnia. Furthermore, the findings suggest the presence of a dose–response relationship. Despite the inherent limitations of this observational study, such as potential residual confounding, and its findings seemingly contradicting the traditional perspective that melatonin possesses beneficial antioxidant and anti-inflammatory properties (32, 33), the identified safety signal merits significant clinical consideration. It is important to note that the randomized controlled trials (RCTs) included in our analysis had a maximum intervention duration of 12 months and primarily concentrated on outcomes related to bone health and quality of life; none were specifically designed to systematically evaluate cardiovascular endpoints. Therefore, the existing RCT evidence is inadequate for drawing definitive conclusions regarding long-term cardiovascular risks. Considering the current body of evidence, it is recommended that clinicians adopt a prudent approach in practice. For patients necessitating prolonged melatonin therapy, especially those with existing cardiovascular risk factors, it is imperative to meticulously balance the potential therapeutic benefits against the indeterminate risks. Treatment should adhere to the principle of employing the minimal effective dose for the briefest duration required, with intensified monitoring throughout the treatment period. Future research endeavors should focus on conducting rigorously designed, prospective randomized controlled trials to specifically examine the long-term cardiovascular effects associated with various melatonin dosages and formulations in distinct patient populations.

The overall certainty of the evidence for all outcomes, evaluated using the GRADE framework (refer to Supplementary Table S1), was determined to be low or very low. This assessment was primarily attributed to significant imprecision, inconsistency, and indirectness.

## Conclusion

This systematic review and meta-analysis suggest that melatonin-containing supplements may be associated with improved bone mineral density (BMD) in menopausal women, with a more consistent signal observed at the femoral neck. It is important to acknowledge that all studies reporting on bone mineral density (BMD) outcomes utilized combination therapies, such as those including calcium and vitamin D, thereby precluding a definitive evaluation of the independent effects of melatonin. Available evidence from limited trials indicates that doses of 3 mg or higher may positively affect bone density. However, the considerable heterogeneity in dosage (1 mg to 3 g) and the prevalent use of combination therapies in the included trials preclude definitive conclusions regarding an optimal dose or the independent effect of melatonin. Our meta-analysis did not identify statistically significant benefits of melatonin supplementation for various health outcomes, including menopause-related symptoms, sleep patterns, emotional well-being, sexual function, insulin levels, and body mass index (BMI). Nevertheless, due to the imprecision of the estimates, as indicated by wide confidence intervals, and the considerable heterogeneity among the studies, these findings should be regarded as inconclusive rather than definitive evidence of the absence of an effect. Future research should prioritize well-designed trials, particularly those incorporating a three-arm design (e.g., melatonin alone, combination therapy, and placebo) to clarify the independent contribution of melatonin. Additionally, long-term studies are needed to evaluate the effect of melatonin on fracture incidence in high-risk populations.

## Data Availability

The original contributions presented in the study are included in the article/Supplementary material, further inquiries can be directed to the corresponding author.
