# Supplementary material for: A systematic review and meta-analysis of randomized controlled trials investigated the effects of melatonin supplementation on bone mineral density, quality of life, and sleep in menopausal women
Source: Front Nutr. 2026 Jan 29;13:1687221. doi: 10.3389/fnut.2026.1687221 (PMC12894000; doi:10.3389/fnut.2026.1687221)
Supplement: Supplementary file 1 [file Table_1.docx]

Table S1. GRADE Evidence Quality Summary Table

| **Outcome** | **Population/Intervention** | **Number of RCTs** | **Sample Size** | **Effect Estimate (95% CI)** | **Heterogeneity (I²)** | **Evidence Quality** | **Key GRADE Judgments** |
| --- | --- | --- | --- | --- | --- | --- | --- |
| **Primary Outcome** |  |  |  |  |  |  |  |
| Bone Mineral Density (Femoral Neck) | Postmenopausal/Perimenopausal women; Melatonin (1-10 mg/d) ± bone micronutrients vs. Placebo | 2 | 123 | Significant increase (P<0.01 to <0.001) | Not applicable (no pooling) | Moderate | + Consistent effect direction; + Dose-response relationship; - Small sample size; - Combination therapy confounding |
| Bone Mineral Density (Lumbar Spine) | Postmenopausal/Perimenopausal women; Melatonin (1-10 mg/d) ± bone micronutrients vs. Placebo | 2 | 123 | Mixed (P=0.04 to 0.021) | Not applicable (no pooling) | Low | - Inconsistent study results; - Small sample size; - Measurement tool heterogeneity |
| **Secondary Outcomes** |  |  |  |  |  |  |  |
| Sleep Quality | Postmenopausal/Perimenopausal women; Melatonin (1-10 mg/d) vs. Placebo | 3 | 81 | SMD -0.87 (-1.94 to 0.19), P=0.11 | 84% | Very Low | - High heterogeneity; - Small sample size; - Variable measurement tools (PSQI/ISI) |
| Menopausal Symptoms | Postmenopausal/Perimenopausal women; Melatonin (3-8 mg/d) vs. Placebo | 3 | 73 | SMD -0.71 (-2.15 to 0.72), P=0.33 | 0% | Low | - Small sample size; - Wide confidence interval (includes clinical benefit) |
| Anxiety | Postmenopausal women; Melatonin (3-8 mg/d) vs. Placebo | 2 | 86 | SMD 0.24 (-0.18 to 0.67), P=0.26 | 0% | Moderate | + Low heterogeneity; - Small sample size; + Standardized measurement tools (STAI/HARS) |
| Depression | Postmenopausal women; Melatonin (3-8 mg/d) vs. Placebo | 2 | 86 | SMD 0.26 (-0.17 to 0.68), P=0.236 | 0% | Moderate | + Low heterogeneity; - Small sample size; + Consistent study results |
| Sexual Function | Postmenopausal women; Melatonin (3 mg/d) vs. Placebo | 2 | 131 | SMD 0.15 (-0.84 to 1.14), P=0.77 | 81% | Very Low | - High heterogeneity; - Sample size imbalance; - Complex scoring scales |
| BMI | Postmenopausal women; Melatonin (1-8 mg/d) vs. Placebo | 3 | 86 | SMD -0.42 (-1.26 to 0.43), P=0.33 | 84% | Very Low | - High heterogeneity; - Variable doses (1-8 mg/d); - No adjustment for body composition |
| Serum Insulin Levels | Postmenopausal/Perimenopausal women; Melatonin (3-3000 mg/d) vs. Placebo | 2 | 56 | SMD -0.01 (-0.38 to 0.36), P=0.97 | 0% | Low | - Small sample size; - Extreme dose variability (3 mg-3 g) |
| Safety (Adverse Events) | Postmenopausal women; Melatonin (3-10 mg/d) vs. Placebo | 2 | 141 | OR 1.93 (0.60 to 6.21), P=0.27 | 0% | Low | - Few studies reporting safety data; - Short follow-up (≤12 months); - No serious adverse events reported |

GRADE Quality Definitions:

High: Further research is very unlikely to change our confidence in the estimate of effect.

Moderate: Further research is likely to have an important impact on our confidence in the estimate of effect and may change the estimate.

Low: Further research is very likely to have an important impact on our confidence in the estimate of effect and is likely to change the estimate.

Very Low: Any estimate of effect is very uncertain.
