# Supplementary material for: A systematic review and meta-analysis of randomized controlled trials investigated the effects of melatonin supplementation on bone mineral density, quality of life, and sleep in menopausal women
Source: Front Nutr. 2026 Jan 29;13:1687221. doi: 10.3389/fnut.2026.1687221 (PMC12894000; doi:10.3389/fnut.2026.1687221)
Supplement: Supplementary file 2 [file Data_Sheet_1.docx]

**Supplementary materials**

**S1 Search strategy**

**Search strategy for PubMed**

**advanced search：**

**#1 ((Melatonin[MeSH Terms]) OR (Melatonin[Title/Abstract])) OR (N-acetyl serotonin[Title/Abstract])**

**#2 ((((Menopause[MeSH Terms]) OR (Menopause[Title/Abstract])) OR (post-menopause[Title/Abstract])) OR (peri-menopause[Title/Abstract])) OR (climacteric[Title/Abstract])**

**#3 ("2015/01/01"[Date - Publication] : "2024/12/31"[Date - Publication])**

**#4 #1 AND #2 AND #3**

**Search strategy for Web of Science**

**advanced search：**

**#1 (((TS=(Menopause)) OR TS=(post-menopause)) OR TS=(peri-menopause)) OR TS=(climacteric)**

**#2 (TS=(Melatonin)) OR TS=(N-acetyl serotonin)**

**#3 #1 AND #2 Publication Date:2015-01-01-2024-12-31**

**Search strategy for Embase**

**advanced search：**

**#1 'Melatonin' OR 'N-acetyl serotonin'**

**#2 'Menopause' OR 'post-menopause' OR 'peri-menopause' OR 'climacteric'**

**#3 #1 AND #2 AND 2015-2024**

**S2 Supplementary Figure S1 Summary of bias risk**


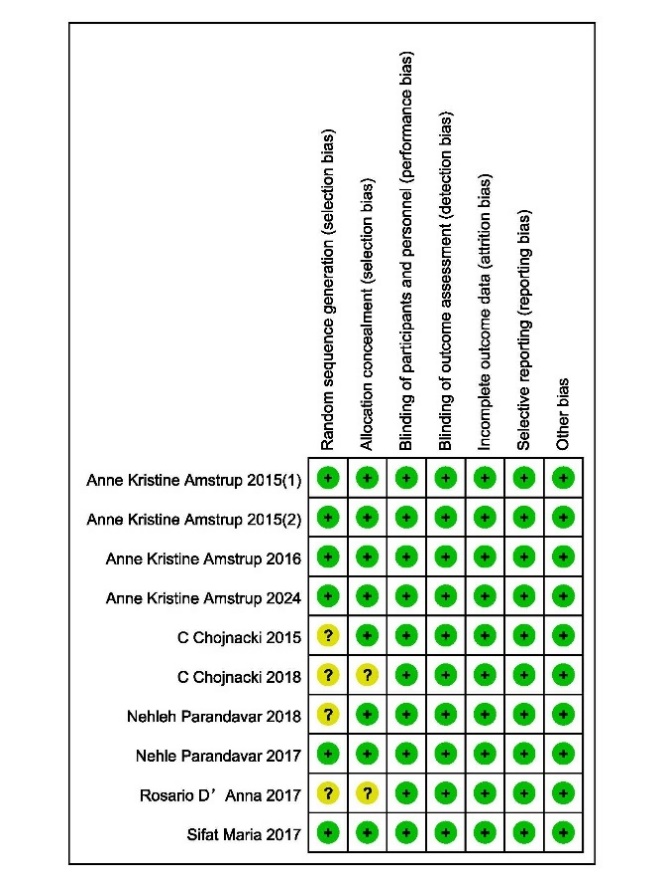


Fig.S1 Summary of bias risk
